# Regional disparities and their contribution to the coverage of the tetanus toxoid vaccine among women aged 15–49 years in Indonesia

**DOI:** 10.12688/f1000research.53004.2

**Published:** 2021-08-31

**Authors:** Hidayat Arifin, Restuning Widiasih, Rifky Octavia Pradipta, Yulia Kurniawati

**Affiliations:** 1 Department of Medical-Surgical Nursing, Faculty of Nursing, Universitas Padjadjaran, Bandung, Indonesia; 2 Department of Maternity Nursing, Faculty of Nursing, Universitas Padjadjaran, Bandung, Indonesia; 3 Faculty of Nursing, Universitas Airlangga, Surabaya, Indonesia

**Keywords:** vaccine; tetanus toxoid; tetanus vaccine; demographic health survey, women

## Abstract

**Background:** The prevention of *Clostridium tetani* bacterial infection through the administration of the tetanus toxoid (TT) vaccine in women is important. The purpose of this study was to determine the regional disparities and factors associated with TT vaccine coverage in women aged 15–49 years in Indonesia.

**Methods: **The Indonesian Demographic Health Survey (IDHS) 2017 data was used in this study. A total of 36,028 women, aged 15–49 years were recruited using the two-stage stratified cluster sampling technique. The questionnaire used was based on the DHS Questionnaire Phase 7. Chi-squared and binary logistic regression were used in this study as part of the analysis.

**Results:** We found that the TT vaccine coverage was 75.32% and that the majority were spread across several provinces. The provinces of Bali and Nusa Tenggara, the richer respondents, living in a rural area, visiting the health facility, having health insurance, and those currently working were factors making it more likely that the women would receive the TT vaccine. The respondents aged 15–24 years with a primary education level and the respondents who were divorced were less likely to receive the TT vaccine.

**Conclusion:** The coverage of the TT vaccine among women can be increased by considering the regional disparities in Indonesia and the socio-economic demographic details of the respondents. Strengthening the policies from the central government in the local governments can improve the screening process and vaccine delivery outcomes. In addition, the importance of giving the TT vaccine to women needs to be relayed through health education in collaboration between health workers and the public.

## Introduction

Neonatal tetanus (NT) is a disease that can be prevented. It has become a global health problem with both high case and high fatality rates among neonates. ^1^ NT refers to tetanus that occurs at 28 days of early life. ^2^ NT occurs due to the toxins produced by *Clostridium tetani* alongside an unhygienic labor agent. It spreads through the umbilical cord. ^3^ One of the efforts to prevent the incidence of NT is the provision of an adequate tetanus toxoid (TT) vaccine for women of reproductive age. ^4^ In 2006, WHO developed guidelines for the TT vaccine for pregnant women to prevent NT. ^5^ The Government of Indonesia through the regulation of the Minister of Health no 12 of 2017 concerning the implementation of immunization stipulates a national immunization coverage target of at least 90% and in districts/cities of at least 80% for TT vaccination as continued vaccine. ^6^

Two doses of the TT vaccine can provide immunity and reduce neonatal mortality by 94%. ^7, 8^ One case–control study also reported that the administration of two doses of the TT vaccine was associated with a decrease in the incidence of NT ^9, 10^ and *vice versa.* ^11^ Minimum two doses of TT vaccine were identified can reduce neonatal mortality. ^12^ TT vaccination contributes to the reduction of neonatal mortality due to tetanus by 85% from 2000 to 2018. ^1^ The Indonesian Health Profile data from the years 2013–2015 show the trend that not receiving the TT vaccine is the leading cause of neonatal mortality. ^13– 15^ Although NT can be prevented by the TT vaccine, the number of cases is still high. Globally, it is estimated that as many as 3.6 million neonates die every year, among which 59,000 die from tetanus. ^16^ The infant mortality rate in Indonesia according to the Indonesian Demographic and Health Survey in 2007 was 34 deaths per 1,000 live births with the highest number of deaths occurring during the neonatal period. ^17^ The neonatal mortality rate in Indonesia in 2007 was 19 per 1000 live births and NT was one of the main causes of death. ^18, 19^

Neonatal tetanus cases still cannot be handled completely because the coverage of the TT vaccine is still lacking, both nationally and regionally. Nationally, the coverage of the TT vaccine for both pregnant and reproductive age women tends to fluctuate and has not yet reached the target. The TT vaccine coverage for pregnant and reproductive age women from 2007 to 2011 was 26% and 27.1%, 65.2% and 24.7%, 73.5% and 11.2%, 69.5% and 8, 6%, and 63.6% and 11.8%, respectively. ^17^ If the regional data is examined randomly, the coverage of the TT vaccine in select regions has also not reached the target. ^20^ The difference in coverage from the highest and lowest during the period was possible due to differences in perceptions of the operational definition, problems in recording, reporting formats, and others. So there needs to be an effort to organize a recording and reporting system for pregnant women TT vaccination. ^17^

The low coverage of the TT vaccine is largely influenced by inadequate knowledge. ^21, 22^ Women with poor knowledge of MNT and TT vaccine are 0.435 times less likely to receive the TT vaccine. ^23^ Insufficient knowledge of the TT vaccine among prospective brides of reproductive age are one of the factors for the low TT vaccine coverage. ^24^ Based on the data above, to overcome this inadequate knowledge, health education about TT is needed. Good health education pays attention to and identifies people’s characteristics in the intended category so then the health education provided can be more effective. ^25^ This study is focused on revealing TT vaccine coverage and the determinant factors of the TT vaccine being received in relation to women of reproductive age.

Several previous Indonesian studies have revealed the coverage and determinants of the TT vaccine. Research into the TT coverage is still reported on a regional scale. ^26, 27^ Research on the determinants of the vaccine for women of childbearing age specifically includes knowledge, family support, attitudes, and the behavior of the health workers. ^22, 28, 29^ This study used available national data including age, education level, wealth quintile, residence, marital status, visiting the health facilities, health insurance, occupational status, the sex of the household head, pregnancy, and the different regional areas in relation to the TT vaccine. It is expected that the results of this study can be used when devising effective approaches for the education of women of reproductive age to promote the TT vaccine.

## Methods

### Study design

A cross-sectional study design was undertaken. We used secondary data from the Indonesian Demographic Health Survey (IDHS) 2017 and parts of the Inner-City Fund (ICF) International data.

### Sample

The survey was conducted in December 2017. We used the IDIR71FL dataset (Indonesian Individual Recode phase 7). The total study population was 49,627 women aged 15–49 years. We then weighted the data based on the number of provinces in Indonesia in order to obtain the average for each region. We managed to reach 36,028 women aged 15–49 years who have not received the TT vaccine in Indonesia. Furthermore, there was missing data. Two-stage stratified cluster sampling was used in this study by selecting clusters from each stratum and a list of families from the selected clusters. Then the families’ questionnaire responses were investigated (Demographic Health Survey, 2017).

### Variables

The independent variables in this study included age, education level, wealth quintile, residence, marital status, visiting the health facility, health insurance, occupational status, sex of household head, pregnancy, regional disparities. Age was categorized into 15–24 years old, 25–34 years old and 35–49 years old (Health Ministry of Republic Indonesia, 2009). Based on Law No. 20 of 2003 concerning the National Education System in Indonesia, education level was categorized into high, secondary, primary, and no education. ^30^ The wealth quintiles were divided into five categories based on principal component analysis (PCA). The wealth quintiles were measured by the percentage distribution of the de jure population using the wealth quintiles and the Gini coefficient. For the percentage distribution, the numerators were divided by the denominators multiplied by 100. The results were then divided into five equal parts from quintile one (poorest) to quintile five (richest); each included 20% of the total population. Then, it was categorized into poorest, poorer, middle, richer, and richest respectively. ^31, 32^ Residence was categorized into either an urban or rural area. ^33^ Marital status was classified as either married, partnered, widowed, divorced or separated. The respondents who had visited a health facility in the last six months, whether they had health insurance, if they were currently working, and their pregnancy status were all categorized as either yes or no. ^31^ We identified the sex of the household head as either male or female. For the regions in Indonesia, we classified the country based on the big islands as follows: Sumatera, Riau, Java, Bali and Nusa Tenggara, Kalimantan, Sulawesi, Maluku, and Papua. ^34^

The dependent variable in this study was the TT vaccine. We identified women aged 15–49 years who either received or did not receive the TT Vaccine. Then we categorized them according to their answer of either yes or no (Demographic Health Survey, 2017). To enhance the quality and transparency of reporting the study results, the researchers applied The Strengthening the Reporting of Observational Studies in Epidemiology (STROBE). ^35^

### Data analysis

We used the STATA version 16.1: “A Software resource for statistical analysis and presentation of graphics (Stata, RRID:SCR_012763)”. We used Chi-squared to analyze the bivariate data and binary logistic regression to analyze the multivariate data. We used the adjusted odds ratio (AOR) with a 95% confidence interval (CI) and a significance level of p < 0.05.

### Ethical considerations

Ethical approval for the secondary dataset was not required. The dataset policy is available on the official website. We received approval to use the dataset from ICF International with number AuthLetter_154679.

## Results

In this study, we found that the coverage of the TT vaccine in Indonesia reached 75.32% out of the 36,028 respondents. More than half of the total respondents who received the vaccine aged 35–49 years were educated to secondary school level. The distribution of wealth quintiles was almost the same, ranging from poorest to richest. The majority were in the poorest quintile. In addition, the distribution of residence was almost the same across both urban and rural areas. The majority of the respondents in this study were married and they had regularly visited health facilities in the last six months. We found that the majority of respondents had health insurance, were working, and were not pregnant. The majority of the household heads were male.

Upon examining the regional distribution data in Indonesia, the TT vaccine coverage was more than 70% in Riau, Java, Bali and Nusa Tenggara, Kalimantan, Sulawesi, and Papua. The majority of respondents aged 35–49 years were spread across Indonesia. The distribution data indicate that the majority of respondents were educated to secondary school level, followed by those with primary education. The majority of respondents in the richest quintile and living in an urban area in Riau and Java. The majority of respondents in the poorest quintile and living in a rural area were in Bali and Nusa Tenggara, Sulawesi, Maluku, and Papua. Most of the respondents were married, had health insurance, were working, were not pregnant, and the household head was male. The respondents in Riau, Maluku, and the Papua islands responded stating that they had rarely visited the health facilities in the last six months ( Table 1).

**Table 1.** Socio-demographic characteristic based on region (n = 36,028).

Variable	n	%	Region															
			Sumatera (n = 8,344)		Riau (n = 753)		Java (n = 12,336)		Bali & Nusa Tenggara (n = 3,053)		Kalimantan (n = 3,291)		Sulawesi (n = 5,415)		Maluku (n = 1,945)		Papua (n = 891)	
			n	%	n	%	n	%	n	%	n	%	n	%	n	%	n	%
TT vaccine No Yes	8,893 27,135	24.68 75.32	2,618 5,726	31.38 68.62	199 554	26.43 73.57	3,157 9,179	25.59 74.41	499 2,554	16.34 83.66	701 2,590	21.30 78.70	898 4,517	16.58 83.42	608 1,337	31.26 68.74	213 678	23.91 76.09
Age 35–49 years old 25–34 years old 15–24 years old	19,697 12,325 4,006	54.67 34.21 11.12	4,540 2,955 849	54.41 35.41 10.17	448 253 52	59.50 33.60 6.91	7,077 3,970 1,289	57.37 32.18 10.45	1,649 1,060 344	54.01 34.72 11.27	1,678 1,216 397	50.99 36.95 12.06	2,910 1,812 693	53.74 33.46 12.80	987 700 258	50.75 35.99 13.26	408 359 124	45.79 40.29 13.92
Education level High education Secondary education Primary education No education	5,251 18,770 11,254 753	14.57 52.10 31.24 2.09	1,321 4,550 2,323 150	15.83 54.53 27.84 1.80	118 475 152 8	15.67 63.08 20.19 1.06	1,467 6,587 4,127 155	11.89 53.40 33.45 1.26	431 1,349 1,122 151	14.12 44.19 36.75 4.95	429 1,719 1,075 68	13.04 52.23 32.66 2.07	960 2,579 1,754 122	17.73 47.63 32.39 2.25	378 1,074 477 16	19.43 55.22 24.52 0.82	147 437 224 83	16.50 49.05 25.14 9.32
Wealth quintiles Poorest Poorer Middle Richer Richest	8,374 7,016 6,945 6,952 6,741	23.24 19.47 19.28 19.30 18.71	1,730 1,908 1,820 1,584 1,302	20.73 22.87 21.81 18.98 15.60	36 77 152 207 281	4.78 10.23 20.19 27.49 37.32	1,145 2,047 2,594 3,168 3,382	9.28 16.59 21.03 25.68 27.42	1,477 543 364 335 334	48.38 17.79 11.92 10.97 10.94	671 683 774 609 554	20.39 20.75 23.52 18.51 16.83	1,891 1,216 862 714 732	34.92 22.46 15.92 13.19 13.52	995 376 259 222 93	51.16 19.33 13.32 11.41 4.78	429 166 120 113 63	48.15 18.63 13.47 12.68 7.07
Residence Urban Rural	18,316 17,712	50.84 49.16	3,478 4,866	41.68 58.32	680 73	90.31 9.69	8,228 4,108	66.70 33.30	1,212 1,841	39.70 60.30	1,729 1,562	52.54 47.46	1,978 3,437	36.53 63.47	774 1,171	39.79 60.21	237 654	26.60 73.40
Marital status Married Partner Widowed Divorced Separated	33,294 369 884 1,319 162	92.41 1.02 2.45 3.66 0.45	7,816 5 217 294 12	93.67 0.06 2.60 3.52 0.14	698 2 18 35 0	92.70 0.27 2.39 4.65 0.00	11,542 12 281 472 29	93.56 0.10 2.28 3.83 0.24	2,677 169 84 78 45	87.68 5.54 2.75 2.55 1.47	3,068 6 78 136 3	93.22 0.18 2.37 4.13 0.09	5,016 19 128 235 17	92.63 0.35 2.36 4.34 0.31	1,764 61 46 49 25	90.69 3.14 2.37 2.52 1.29	713 95 32 20 31	80.02 10.66 3.59 2.24 3.48
Visiting health facility No Yes	16,639 19,389	46.18 53.82	3,751 4,593	44.95 55.05	384 369	51.00 49.00	5,544 6,792	44.94 55.06	1,406 1,647	46.05 53.95	1,454 1,837	44.18 55.82	2,529 2,886	46.70 53.30	1,066 879	54.81 45.19	505 386	56.68 43.32
Health insurance No Yes	13,839 22,189	38.41 61.59	3,080 5,264	36.91 63.09	275 478	36.52 63.48	5,032 7,304	40.79 59.21	1,174 1,879	38.45 61.55	1,475 1,816	44.82 55.18	1,732 3,683	31.99 68.01	853 1,092	43.86 56.14	218 673	24.47 75.53
Currently working No Yes	14,978 21,050	41.57 58.43	3,393 4,951	40.66 59.34	335 418	44.49 55.51	5,478 6,858	44.41 55.59	1,054 1,999	34.52 65.48	1,300 1,991	39.50 60.50	2,247 3,168	41.50 58.50	840 1,105	43.19 56.81	331 560	37.15 62.85
Sex of household head Male Female	32,233 3,795	89.47 10.53	7,487 857	89.73 10.27	684 69	90.84 9.16	11,057 1,279	89.63 10.37	2,618 435	85.75 14.25	3,011 280	91.49 8.51	4,847 568	89.51 10.49	1,727 218	88.79 88.79	802 89	90.01 9.99
Pregnancy No Yes	34,081 1,947	94.60 5.40	7,832 512	93.86 6.14	711 42	94.42 5.58	11,766 570	95.38 4.62	2,896 157	94.86 5.14	3,106 185	94.38 5.62	5,139 276	94.90 5.10	1,796 149	92.34 7.66	835 56	93.71 6.29

The bivariate analysis showed that the regional variables, age, education level, wealth quintile, residence, marital status, whether they visited the health facilities, health insurance, whether they were currently working, and the sex of the household head have a significant relationship with TT vaccine coverage in women aged 15–49 years. However, the pregnancy variable did not have a significant relationship with TT vaccine coverage ( Table 2).

**Table 2.** Bivariate analysis of tetanus toxoid vaccine coverage among women aged 15–49 years in Indonesia (n = 36,028).

Variable	Tetanus toxoid vaccine coverage				
	No		Yes		χ ^**2**^
	n	%	n	%	
Regional (island) Sumatera Riau Java Bali & Nusa Tenggara Kalimantan Sulawesi Maluku Papua	2,618 199 3,157 499 701 898 608 213	7.27 0.55 8.76 1.39 1.95 2.49 1.69 0.59	5,726 554 9,179 2,554 2,590 4,517 1,337 678	15.89 1.54 25.48 7.09 7.19 12.54 3.71 1.88	578.813 ***
Age 35–49 years old 25–34 years old 15–24 years old	4,940 2,830 1,123	13.71 7.86 3.12	14,757 9,495 2,883	40.96 26.35 8.00	45.499 ***
Education level High education Secondary education Primary education No education	914 3,879 3,631 469	2.54 10.77 10.08 1.30	4,337 14,891 7,623 284	12.04 41.33 21.16 0.79	1,200 ***
Wealth quintiles Poorest Poorer Middle Richer Richest	2,710 1,756 1,627 1,461 1,339	7.52 4.87 4.52 4.06 3.72	5,664 5,260 5,318 5,491 5,402	15.72 14.60 14.76 15.24 14.99	406.478 ***
Residence Urban Rural	4,220 4,673	11.71 12.97	14,096 13,039	39.13 36.19	54.138 ***
Marital status Married Partner Widowed Divorced Separated	7,978 113 313 430 59	22.14 0.31 0.87 1.19 0.16	25,316 256 571 889 103	70.27 0.71 1.58 2.47 0.29	127.472 ***
Visiting health facility No Yes	5,108 3,785	14.18 10.51	11,531 15,604	32.01 43.31	601.783 ***
Health insurance No Yes	3,865 5,028	10.73 13.96	9,974 17,161	27.68 47.63	127.253 ***
Currently working No Yes	3,911 4,982	10.86 13.83	11,067 16,068	30.72 44.60	28.121 ***
Sex of household head Male Female	7,825 1,068	21.72 2.96	24,408 2,727	67.75 7.57	27.295 ***
Pregnancy No Yes	8,418 475	23.37 1.32	25,663 1,472	71.23 4.09	0.091

*** p < 0.01.

** p < 0.05.

* p < 0.1.

χ ^2^: Chi-squared.

Table 3 shows the results of the multivariate analysis. The data indicate that regional disparities, age, education level, wealth quintile, residence, marital status, whether they had visited the health facilities recently, and having health insurance are likely to be associated with TT vaccine coverage in women aged 15–49 years in Indonesia. The regional data shows that the respondents in Bali and Nusa Tenggara are 3.363-times more likely to receive the TT vaccine than the respondents in Sumatera (AOR = 3.363; 95%CI = 2.997–3.773). The respondents aged –24 years old are 0.71-times less likely to receive the TT vaccine than those aged 35–49 years (AOR = 0.71; 95%CI = 0.653–0.772). Regarding education level, the respondents with a primary school level of education were 0.544-times less likely to receive the TT vaccine than the respondents with a higher level of education (AOR = 0.544; 95%CI = 0.494–0.599). Furthermore, the richer respondents were 1.645-times more likely to receive the TT vaccine than the poorest respondents (AOR = 1.645; 95%CI = 1.506–1.798). The respondents living in rural areas were 1.106-times more likely to have had the TT vaccine than those living in urban areas (AOR = 1.106; 95%CI = 1.044–1.173). Divorced respondents were 0.693-times less likely to receive the TT vaccine than married respondents (AOR = 0.693; 95%CI = 0.608–0.79). The respondents who had regularly visited a health facility in the last six months were 1.693-times more likely to receive the vaccine than those who had not (AOR = 1.693; 95%CI = 1.609–1.781). The respondents who had health insurance were 1.176-times more likely to receive the vaccine than those who did not (AOR = 1.176; 95%CI = 1.117–1.239). The respondents who worked were 1.147-times more likely to receive the vaccine than those who did not (AOR = 1.147; 95%CI = 1.088–1.208).

**Table 3.** Multivariate analysis of tetanus toxoid vaccine coverage among women aged 15–49 years in Indonesia (n = 36,028).

Variable	Tetanus toxoid vaccine coverage			
	AOR	p	95%CI	
			Lower	Upper
Regional (island) Sumatera Riau Java Bali & Nusa Tenggara Kalimantan Sulawesi Maluku Papua	*Ref.* 1.197 ** 1.353 *** 3.363 *** 1.874 *** 2.752 *** 1.217 *** 2.247 ***	0.044 0.000 0.000 0.000 0.000 0.001 0.000	1.005 1.266 2.997 1.697 2.517 1.086 1.88	1.427 1.445 3.773 2.07 3.01 1.362 2.686
Age 35–49 years old 25–34 years old 15–24 years old	*Ref.* 0.939 ** 0.71 ***	0.031 0.000	0.886 0.653	0.994 0.772
Education level High education Secondary education Primary education No education	*Ref.* 0.994 0.544 *** 0.149 ***	0.901 0.000 0.000	0.911 0.494 0.125	1.085 0.599 0.179
Wealth quintiles Poorest Poorer Middle Richer Richest	*Ref.* 1.442 *** 1.544 *** 1.645 *** 1.527 ***	0.000 0.000 0.000 0.000	1.334 1.421 1.506 1.382	1.558 1.676 1.798 1.687
Residence Urban Rural	*Ref.* 1.106 ***	0.001	1.044	1.173
Marital status Married Partner Widowed Divorced Separated	*Ref.* 0.691 *** 0.675 *** 0.693 *** 0.585 ***	0.003 0.000 0.000 0.002	0.541 0.573 0.608 0.415	0.882 0.796 0.79 0.824
Visiting health facility No Yes	*Ref.* 1.693 ***	0.000	1.609	1.781
Health insurance No Yes	*Ref.* 1.176 ***	0.000	1.117	1.239
Currently working No Yes	*Ref.* 1.147 ***	0.000	1.088	1.208
Sex of household head Male Female	*Ref.* 0.939	0.177	0.857	1.029

*** p < 0.01.

** p < 0.05.

* p < 0.1.

AOR: Adjusted odds ratio, CI: confidence interval.

## Discussion

In this study, we discussed the gap in the reception of the TT vaccine among women aged 15–49 years in Indonesia by looking at the regional disparities. We also were able to determine the contributions behind the achievement of the TT vaccine coverage in Indonesia as it stands. We found that regional disparities were significantly associated with TT vaccine performance. In addition, the factors of age, education level, wealth quintile, residence, marital status, whether they visited the health facilities, health insurance, and whether they were currently working also contribute to the TT vaccine coverage among women aged 15–49 years in Indonesia.

Regional disparities are one of the demographic factors that contribute greatly to the TT vaccine coverage among women. Indonesia, an archipelago region, can be an inhibiting factor regarding vaccine coverage. ^36, 37^ This geographical condition can affect the coverage in vaccination because it affects the access, availability, distribution of the vaccine supply chain between islands. Differences in culture, region, ethnicity, language, knowledge, and access in Indonesia are important factors to consider when seeking to facilitate access to get a vaccine. ^38, 39^ According to this study, the Bali and Nusa Tenggara regions have a greater chance of administering the TT vaccine to their respective populations than other regions. When viewed according to socio-economic development, Bali and Nusa Tenggara, which are included within Eastern Indonesia, are far behind compared with the Java, Sumatera and Riau. ^40, 41^ This is consistent with the previous research which states that vaccine coverage can be influenced by region, development, knowledge and awareness of the disease and vaccine. ^42, 43^

We found that the younger respondents, aged 15–24 years, were less likely to receive the TT vaccine than the respondents who were older (35–49 years old). A previous study showed that age is related to the knowledge of the importance of vaccines and the ability to make decisions. ^44, 45^ Therefore, health education is needed among those of a young age about the importance of vaccines. In addition, in this study it is also known that this is less likely for those of a primary education level compared with those with a higher level of education. This is because with a good level of knowledge, TT vaccination coverage can be achieved. In this case, the government and health workers have an important role in distributing knowledge about vaccines to the public. Both previous studies have shown that vaccine performance is influenced by a good level of knowledge. ^46, 47^ Moreover, respondents aged 15-24 years have less experience, beliefs, and family non-supporting about vaccine. ^48, 49^ This condition tendency of less likely to to receive the TT vaccine.

In this study, we found that economic status contributed to the achievement of TT vaccine coverage in women. Based on the finding in this study, respondents with a wealth quintile that is higher and those with a job are more likely to receive the TT vaccine compared with the respondents whose economic level is low and who do not work. Previous research has shown that the respondents with a stable income can easily access private and government health facilities and get vaccines. ^50, 51^ In addition, the respondents who had health insurance were found to be more likely to receive the TT vaccine. This is because the respondent feels calm that their medical costs will be covered by their health insurance. Previous research has shown that with health insurance, vaccine coverage can increase. It was because people should not think about the payment for TT vaccine. ^52– 54^ It can be one of the program from government to reach high vaccination coverage.

In terms of the rural areas, the study found that the residents of these areas are more likely to receive the TT vaccine. This is related to the obedience of the rural population where doctors, nurses and midwives have been able to gain the trust of the community. ^55^ This closeness is also obtained through the routine outreach process used to engage with the community members. ^56^ The information obtained by the rural residents tends to be more centralized and there is no intervention from other sources such as the internet and minimizing false news; the information will be centered on the doctors, nurses and midwives visited at the health facility as a result. ^57, 58^ Also, rural residents rely more on health workers where they live. In this context, it is the midwife who mostly works in remote villages. ^17^ Rural residents trust these midwives and regard the midwife as a link between them and the health care facilities provided by the government. This is in contrast to the urban residents who prefer to obtain their health-related information independently. They tend to compare the results of the information obtained from the internet with that of the doctors, nurses and midwives at the health facilities. The gaps in the information obtained trigger doubts about the TT vaccine and in the end, there is a delay in getting the vaccine even after marriage.

This study found that the women who visit the health facility are more likely to receive the TT vaccine. Visiting the health facility will increase their information and knowledge related to vaccines. ^59^ In addition, each visit can increase their closeness and bond with the health workers. This can convince the women aged 15–49 years old to have the TT vaccine. A visit to a health care facility provides an opportunity for women to clarify and address their concerns regarding the side effects of vaccines by consulting directly with their doctor or nurse. ^60^ Rumors circulating in the community regarding the TT vaccine also can be explained through counselling during the visits. One of the rumors is that the content of the vaccine material is not halal, so there is resistance from some women. Health workers like the doctors and nurses are needed to clarify the problem. ^61^

Those who are divorced are less likely to receive the TT vaccine. This is associated by the decrease or non-existence of motivation from a partner. Women who have lived alone tend to have different views after a divorce. Focus and priorities in life may no longer lead to the fulfillment of health support such as vaccines, but rather to be economically independent and able to meet the needs of daily life. Divorced women do not feel the need to receive the TT vaccine because one of the goals of this vaccine is to reduce the risk of tetanus in women and their unborn baby. ^62^ If they are divorced, there is no need for the TT vaccine. Indonesian government have many efforts to increase the TT vaccination coverage, such as equitable availability of midwives in rural to remote areas ^17^ and regulations that require brides to be vaccinated TT according to government regulation number 02 of 1989 about Tetanus Toxoid Immunization before marriage. ^63^

The strength of our study is that it provides information on the TT vaccine coverage nationally while highlighting the regional disparities in Indonesia. The results of this study can become basic data for the Indonesian government to use to determine further policies to achieve an improved level of TT vaccine coverage among women. However, this study has limitations in that the researchers looked at the distribution based only on the major islands in Indonesia. Descriptions at the provincial level are needed for the formulation of more specific policies. Meanwhile, the study has assumption of no significant correlation among predictors.

## Conclusion

In this study, we found there to be a gap in the TT vaccine uptake among women aged 15–49 years in Indonesia. Indonesia as an archipelago country become one of the considerations and constraints involved in the coverage of the TT vaccine among women. The findings of this study provide an overview about the TT vaccine coverage according to several factors such as the regional disparities and the respondents’ socio-economic demographic information. Furthermore, the government can collaborate across different sectors between the central and local governments to achieve the desired TT vaccine coverage. Providing accurate and precise information about the TT vaccine needs to be promoted by healthcare workers in collaboration with the community through online methods to reach the urban population areas in Indonesia. An in-depth exploration with additional factors and sectors involved is needed in terms of the direction of further research.

## Data availability

Data used in this study is available online from the Indonesian 2017 Demographic and Health Survey (DHS) website under the ‘Individual Recode’ section. Access to the dataset requires registration and is granted only for legitimate research purposes. A guide for how to apply for dataset access is available at: https://dhsprogram.com/data/Access-Instructions.cfm.
