# Supplementary material for: Regional disparities and their contribution to the coverage of the tetanus toxoid vaccine among women aged 15–49 years in Indonesia
Source: F1000Res. 2021 Aug 31;10:437. Originally published 2021 Jun 3. [Version 2] doi: 10.12688/f1000research.53004.2 (PMC8378405; doi:10.12688/f1000research.53004.2)
Supplement: Peer review history

# Peer review: Reviewer response for version 2

**DOI:** 10.5256/f1000research.76877.r93100

**Published:** 2021-09-13

**Authors:** Muhammad Hardhantyo

**Affiliations:** 1 Faculty of Health Science, Universitas Respati Yogyakarta, Yogyakarta, Indonesia; 2 Center for Health Policy and Management, Faculty of Medicine, Public Health and Nursing, Universitas Gadjah Mada, Yogyakarta, Indonesia

The manuscript is well-written, including a description of the approach clearly. The authors have addressed all reviewer recommendations given to improve the article's quality. The findings could contribute to enhancing our understanding of tetanus toxoid vaccination coverage of women aged 15–49 years in Indonesia.

Is the work clearly and accurately presented and does it cite the current literature?

Yes

If applicable, is the statistical analysis and its interpretation appropriate?

Yes

Are all the source data underlying the results available to ensure full reproducibility?

Yes

Is the study design appropriate and is the work technically sound?

Yes

Are the conclusions drawn adequately supported by the results?

Yes

Are sufficient details of methods and analysis provided to allow replication by others?

Partly

Reviewer Expertise:

Health Policy, Epidemiology, Child Health, Maternal Health

I confirm that I have read this submission and believe that I have an appropriate level of expertise to confirm that it is of an acceptable scientific standard.

# Peer review: Reviewer response for version 1

**DOI:** 10.5256/f1000research.56345.r90139

**Published:** 2021-08-19

**Authors:** Azza Mehanna

**Affiliations:** 1 Health Administration and Behavioral Sciences Department, HIPH, Alexandria University, Alexandria, Egypt

**Introduction:**

- Paragraph 1, last line: Reference 6 is not in English, and on opening it, it seemed to lack some important details such as the target year for national and regional vaccine coverage of at least 90% and 80% respectively and it (target year) was not mentioned in the manuscript either.
  Also: "as an advanced vaccine”, what do you mean?
- Paragraph 2, line 4: Reference 12 is rather old, aren’t there more recent updates for these figures?
  Also: "and by 45% after the second dose”, I don’t think this percentage is correct, infant mortality shows more decrease after the second dose.
- Paragraph 2, line 7: Reference 16 is rather old, try to find more recent data.
- Paragraph 2, lines 7-8: you got the data from DHS 2007, isn’t there a more recent DHS? You mentioned DHS 2017 in methodology, why haven’t you used it in your review?
- Paragraph 3, line 1: “There are still cases of NT in line with the coverage of the national and regional TT vaccines not yet reaching the target.” This statement is not clear and needs to be rephrased.
- Paragraph 4, lines 1-2: “Women of reproductive age with knowledge of maternal and neonatal tetanus (MNT)”, you probably mean poor knowledge, please clarify.
- Paragraph 4, line 2: “low TT vaccine are 0.435 times more likely to receive the TT”, what do you mean by low tetanus toxoid vaccine? "0.435 times more likely to receive the TT vaccine": this is not a good interpretation of the odds ratio.
- I found some difficulty checking on some references, e.g references 6 and 27, as they were written in a different language.

**Methods:**

 You need to elaborate more on methods as follows:

- Sample, lines 3-4: “We managed to reach 36,028 women aged 15– 49 years who have not received the TT vaccine in Indonesia”, how did you reach them? Did you give them questionnaires? Please clarify.
- Why haven’t you been able to study the whole population mentioned (49,627)? Please clarify.
- This sample of women (36,028) comprises those who received and those who did not receive the vaccine according to Table 1 and not only those who have not received the vaccine as you mentioned.
- Sample, line 4: “Two-stage stratified cluster sampling was used”, what are the two stages? Stratified according to what?

**Results:**

- Tables 3 and 4: You don’t need to put *P<0.1 in the footnotes.
- Table 3, last row: Is the value “0.091” the correct value of χ2? Please check.
- Table 3: Why have you calculated percent from total not from independent variable?
- Table 4, line 2: “and health insurance”, it is better to say "and having health insurance".

**Discussion:**

- Paragraph 2, line 2: “Indonesia, which is an archipelago region, can be an inhibiting factor regarding vaccine coverage”. This statement needs rephrasing and clarification.
- Paragraph 2, lines 7-9: “However, it can be seen in this study that the respondents' awareness of the importance of the TT vaccine is very high. This is consistent with the previous research which states that vaccine coverage can be influenced by region, development, knowledge and self-awareness”:
   
  - “vaccine coverage can be influenced by region, development”: From the context, it seems that you refer to a direct relationship. However, this contradicts your findings as vaccine coverage in Bali and Nusa Tenggara is high (83.66%), despite having a high percentage of the poorest (48.38%) and a relatively low percentage of the richest (10.94%).
  - "self-awareness”: Self-awareness was not studied in this research and it is a totally different concept, I think you mean awareness of the disease and vaccine.
  - “it can be seen in this study that the respondents' awareness of the importance of the TT vaccine is very high”: Awareness of the vaccine and of its importance was not studied in this research, in fact, awareness of the vaccine and of its importance could just be some of the possible factors underlying high coverage rates. You may point to awareness as an inference not as a finding. Please note that awareness of the vaccine is not the same as awareness of the importance of the vaccine.
- Paragraph 3: Why do you relate your studied variables as age and education to knowledge and you continue referring to it (knowledge) despite not being studied in this research? In fact, many other variables could underlie and explain high coverage, such as: applying a good coverage policy, life experiences and beliefs, culture, physicians’ attitude and behavior regarding vaccine, availability and accessibility of vaccine, and social support/pressure.
- Paragraph 3, line 7: “the previous study”, which study? You put two references not one.
- Paragraph 4, lines 1-2: “It is known that respondents with a wealth quintile that is higher and those with a job are more likely to receive the TT vaccine compared with the respondents whose economic level is low and who do not work”:
   
  - “it is known that": From where? Opposite findings are displayed in other studies, for example, Mehanna A, Ali MH, Kharboush I: Knowledge and health beliefs of reproductive-age women in Alexandria about tetanus toxoid immunization. J Egypt Public Health Assoc. 2020; 95(1): 1–11 (Reference 23).
- Paragraph 4, lines 6-7: “Previous research has shown that with health insurance, vaccine coverage can increase”, this statement may be too simple, it needs rephrasing.
- Paragraph 5: You pointed out that residents of rural areas are more likely to receive the TT vaccine, and you attributed that to their obedience to their doctors and nurses. Well, perhaps there are other justifications; maybe physicians in rural areas - who usually work in health units or hospitals affiliated to the ministry of health (MOH) - are more committed to vaccinate or recommend vaccination to their female clients. Maybe females in urban areas rely more on private physicians who may not always adhere to the instructions and regulations of the MOH regarding vaccination.
- Paragraph 6: You found that women who visited the health facility were more likely to receive the TT vaccine, then you started explaining your finding. You gave a good interpretation, however, some information was repeated, e.g “Routine visits to the health facilities can also overcome obstacles that have previously been a barrier to vaccines, including a lack of knowledge and excessive concern about the side effects of the vaccine”. This is a repetition of what you have just said. You may rephrase this paragraph to explain your point without redundancy.
- Paragraph 7: The same comment as paragraph 6. Please rephrase this paragraph to avoid redundancy.
- Paragraph 7, line 4 “Although the TT vaccine is actually a requirement before marriage takes place”, please identify the regulations and policy of the Ministry of Health in Indonesia regarding TT vaccination for females.
- Paragraph 7, line 5 “vaccine can be missed because of the assumption that you have received the vaccine before”, isn’t there vaccination cards or records to refer to by the health personnel to make sure that the female client has been vaccinated? Is vaccine given only in health facilities affiliated with the MOH or it may be administered in private clinics? Please clarify.
- Paragraph 7, lines 5-6: “There is still the assumption that the vaccine is not necessary”, you need to elaborate a little more on this point and identify the rationale beyond this assumption.

**Conclusion**

- Line 1: “we found there to be a gap in the TT vaccine uptake among women aged –49 years old in Indonesia”, if you mean the age group 35-49 then this conclusion contradicts your findings as this group is the highest in vaccine uptake. If you mean 15-49 then you need to explain your conclusion, especially that your results (Table 2) and your Discussion ("However, it can be seen in this study that the respondents' awareness of the importance of the TT vaccine is very high") referred to the high TT vaccine coverage rates.
- Lines 2-3: “Indonesia as a whole, which is an archipelago, is one of the considerations and constraints involved in the coverage of the TT vaccine among women”, you need to clarify this point a little.

Is the work clearly and accurately presented and does it cite the current literature?

Partly

If applicable, is the statistical analysis and its interpretation appropriate?

Partly

Are all the source data underlying the results available to ensure full reproducibility?

Yes

Is the study design appropriate and is the work technically sound?

Partly

Are the conclusions drawn adequately supported by the results?

Partly

Are sufficient details of methods and analysis provided to allow replication by others?

Partly

Reviewer Expertise:

Behavioral Sciences, Health promotion, Health Education

I confirm that I have read this submission and believe that I have an appropriate level of expertise to confirm that it is of an acceptable scientific standard, however I have significant reservations, as outlined above.

# Response

**Published:** 2021-08-22

**Authors:** Hidayat Arifin

**Affiliations:** Universitas Padjadjaran, Indonesia

Dear Dr. Azza Mehanna,

 Thank you very much for the comments and suggestions. Here we put some revisions based on your suggestions. 

 Paragraph 1, last line: Reference 6 is not in English, and on opening it, it seemed to lack some important details such as the target year for national and regional vaccine coverage of at least 90% and 80% respectively and it (target year) was not mentioned in the manuscript either.

- Thank you for the correction. The Government of Indonesia through the regulation of the Minister of Health no 12 of 2017 concerning the implementation of immunization stipulates a national immunization coverage of at least 90% and immunization coverage in districts/cities of at least 80%.

Also: "as an advanced vaccine”, what do you mean?           

- Thank you for the correction. We mean "continued vaccine".

Paragraph 2, line 4: Reference 12 is rather old, aren’t there more recent updates for these figures?       

- Thank you for the suggestion. We have found and added the more recent data. “Minimum 2 doses of TT vaccine were identified can reduce neonatal mortality”

Also: "and by 45% after the second dose”, I don’t think this percentage is correct, infant mortality shows more decrease after the second dose.           

- Thank you for the correction. In line with the addition of recent data in references 1 and 12 (paragraph 2), we also update this percentage. “TT vaccination contributes to the reduction of neonatal mortality due to tetanus by 85% from 2000 to 2018”.

Paragraph 2, line 7: Reference 16 is rather old, try to find more recent data.        

- Thank you for the suggestion. We have found and added the more recent data. “TT vaccination contributes to the reduction of neonatal mortality due to tetanus by 85% from 2000 to 2018”.

Paragraph 2, lines 7-8: you got the data from DHS 2007, isn’t there a more recent DHS? You mentioned DHS 2017 in methodology, why haven’t you used it in your review?   

- We used 2007 DHS data as an initial overview of TT immunization coverage in the introduction. DHS 2017 data is the data we analyzed, so the review is only written in the method, result, discussion section.

Paragraph 3, line 1: “There are still cases of NT in line with the coverage of the national and regional TT vaccines not yet reaching the target.” This statement is not clear and needs to be rephrased.       

- Thank you for the correction, we have paraphrased the sentence.

Paragraph 4, lines 1-2: “Women of reproductive age with knowledge of maternal and neonatal tetanus (MNT)”, you probably mean poor knowledge, please clarify.     

- Thank you for the correction. “Women with poor knowledge of MNT and TT vaccine”.

Paragraph 4, line 2: “low TT vaccine are 0.435 times more likely to receive the TT”, what do you mean by low tetanus toxoid vaccine? "0.435 times more likely to receive the TT vaccine": this is not a good interpretation of the odds ratio.

- Thank you for the correction. We have revised it to be less likely to receive the TT vaccine.

I found some difficulty checking on some references, e.g references 6 and 27, as they were written in a different language.    

- Thank you for your attention. We used those references because they are related to the statements.

Sample, lines 3-4: “We managed to reach 36,028 women aged 15– 49 years who have not received the TT vaccine in Indonesia”, how did you reach them? Did you give them questionnaires? Please clarify.      

- In this study, we used secondary data from the Indonesian Demographic Health Survey (IDHS) 2017 that is part of the Inner-City Fund (ICF) International data. All the data collection was collected by questionnaire and managed by IDHS. Thus, we managed to reach 36,028 women aged 15– 49 years who have and have not received the TT vaccine in Indonesia.

Why haven’t you been able to study the whole population mentioned (49,627)? Please clarify.

- We were not the whole population to be analyzed because we found many missing data and should be excluded. We weighted the data set to make sure the equal portion in each province of Indonesia. Then, we used syntax “keep if” in STATA to exclude the missing data from the analysis.

This sample of women (36,028) comprises those who received and those who did not receive the vaccine according to Table 1 and not only those who have not received the vaccine as you mentioned.        

- Thank you, we have revised it. We managed to reach 36,028 women aged 15– 49 years who received and did not receive the TT vaccine in Indonesia.

Sample, line 4: “Two-stage stratified cluster sampling was used”, what are the two stages? Stratified according to what?         

- The sample was obtained through a two-stage stratified cluster sampling technique including select clusters from each stratum and a list of households in selected clusters. The researchers then selected the households to be interviewed.

Tables 3 and 4: You don’t need to put *P<0.1 in the footnotes.      

- Thank you for the correction.

Table 3, last row: Is the value “0.091” the correct value of χ2? Please check.         

- Yes, the analysis is correct based on chi-square analysis.

Table 3: Why have you calculated percent from total not from independent variable?     

- Because we would like to know the distribution of the percentage of each category.

Table 4, line 2: “and health insurance”, it is better to say "and having health insurance". 

- Thank you for the correction.

Paragraph 2, line 2: “Indonesia, which is an archipelago region, can be an inhibiting factor regarding vaccine coverage”. This statement needs rephrasing and clarification.  

- Thank you for your suggestion. Indonesia is a country that has many islands. This geographical condition can affect the coverage in vaccination because it affects the access, availability, distribution of the vaccine supply chain between islands.

Paragraph 2, lines 7-9: “However, it can be seen in this study that the respondents' awareness of the importance of the TT vaccine is very high. This is consistent with the previous research which states that vaccine coverage can be influenced by region, development, knowledge and self-awareness”: “vaccine coverage can be influenced by region, development”: From the context, it seems that you refer to a direct relationship. However, this contradicts your findings as vaccine coverage in Bali and Nusa Tenggara is high (83.66%), despite having a high percentage of the poorest (48.38%) and a relatively low percentage of the richest (10.94%).

- Thank you for the correction. We have deleted the sentence because of incoherence with the previous sentences.

"self-awareness”: Self-awareness was not studied in this research and it is a totally different concept, I think you mean awareness of the disease and vaccine.   

- Thank you for the correction.

“it can be seen in this study that the respondents' awareness of the importance of the TT vaccine is very high”: Awareness of the vaccine and of its importance was not studied in this research, in fact, awareness of the vaccine and of its importance could just be some of the possible factors underlying high coverage rates. You may point to awareness as an inference not as a finding. Please note that awareness of the vaccine is not the same as awareness of the importance of the vaccine.         

- Thank you for the correction. We have deleted the sentence because of incoherence with the previous sentences.

Paragraph 3: Why do you relate your studied variables as age and education to knowledge and you continue referring to it (knowledge) despite not being studied in this research? In fact, many other variables could underlie and explain high coverage, such as: applying a good coverage policy, life experiences and beliefs, culture, physicians’ attitude and behavior regarding vaccine, availability and accessibility of vaccine, and social support/pressure.      

- We discussed age, knowledge, and level of education because we found many references about it. Moreover, we added that less experience, beliefs, and support from family are also related in this case.

Paragraph 3, line 7: “the previous study”, which study? You put two references not one.

- We mean both previous studies.

Paragraph 4, lines 1-2: “It is known that respondents with a wealth quintile that is higher and those with a job are more likely to receive the TT vaccine compared with the respondents whose economic level is low and who do not work”: “it is known that": From where? Opposite findings are displayed in other studies, for example, Mehanna A, Ali MH, Kharboush I: Knowledge and health beliefs of reproductive-age women in Alexandria about tetanus toxoid immunization. J Egypt Public Health Assoc. 2020; 95(1): 1–11 (Reference 23). 

- We wrote the sentence referring to the result of our study.

Paragraph 4, lines 6-7: “Previous research has shown that with health insurance, vaccine coverage can increase”, this statement may be too simple, it needs rephrasing.  

- Previous research has shown that with health insurance, vaccine coverage can increase. It was because people should not think about the payment for the TT vaccine. It can be one of the programs from the government to reach high vaccination coverage.

Paragraph 5: You pointed out that residents of rural areas are more likely to receive the TT vaccine, and you attributed that to their obedience to their doctors and nurses. Well, perhaps there are other justifications; maybe physicians in rural areas - who usually work in health units or hospitals affiliated to the ministry of health (MOH) - are more committed to vaccinate or recommend vaccination to their female clients. Maybe females in urban areas rely more on private physicians who may not always adhere to the instructions and regulations of the MOH regarding vaccination.   

- Thank you for the suggestion. We have elaborated the sentence. Rural residents rely more on health workers where they live. In this context, it is the midwife who mostly works in remote villages. Rural residents trust these midwives and regard the midwife as a link between them and the health care facilities provided by the government.

Paragraph 6: You found that women who visited the health facility were more likely to receive the TT vaccine, then you started explaining your finding. You gave a good interpretation, however, some information was repeated, e.g “Routine visits to the health facilities can also overcome obstacles that have previously been a barrier to vaccines, including a lack of knowledge and excessive concern about the side effects of the vaccine”. This is a repetition of what you have just said. You may rephrase this paragraph to explain your point without redundancy. 

- Thank you for your suggestion. We have paraphrased the sentence. This study found that the women who visit the health facility are more likely to receive the TT vaccine. Visiting the health facility will increase their information and knowledge related to vaccines. In addition, each visit can increase their closeness and bond with the health workers. This can convince the women aged 15–49 years old to have the TT vaccine. A visit to a health care facility provides an opportunity for women to clarify and address their concerns regarding the side effects of vaccines by consulting directly with their doctor or nurse. Rumors circulating in the community regarding the TT vaccine also can be explained through counselling during the visits. One of the rumors is that the content of the vaccine material is not halal, so there is resistance from some women. Health workers like the doctors and nurses are needed to clarify the problem.

Paragraph 7: The same comment as paragraph 6. Please rephrase this paragraph to avoid redundancy. 

- Thank you for your suggestion. We have paraphrased the sentence. Those who are divorced are less likely to receive the TT vaccine. This is related to the decrease or absence of motivation from the partner. Women who have lived alone tend to have different views after a divorce. Focus and priorities in life may no longer lead to the fulfillment of health support such as vaccines, but rather to be economically independent and able to meet the needs of daily life.

Paragraph 7, line 4 “Although the TT vaccine is actually a requirement before marriage takes place”, please identify the regulations and policy of the Ministry of Health in Indonesia regarding TT vaccination for females.  

- Thank you, we have deleted the sentences because they are not relevant to the previous sentences.

Paragraph 7, line 5 “vaccine can be missed because of the assumption that you have received the vaccine before”, isn’t there vaccination cards or records to refer to by the health personnel to make sure that the female client has been vaccinated? Is vaccine given only in health facilities affiliated with the MOH or it may be administered in private clinics? Please clarify.          

- Thank you, we have deleted the sentences because they are not relevant to the previous sentences.

Paragraph 7, lines 5-6: “There is still the assumption that the vaccine is not necessary”, you need to elaborate a little more on this point and identify the rationale beyond this assumption.        

- Thank you for your correction, we have rewritten the sentence.

           

 Line 1: “we found there to be a gap in the TT vaccine uptake among women aged –49 years old in Indonesia”, if you mean the age group 35-49 then this conclusion contradicts your findings as this group is the highest in vaccine uptake. If you mean 15-49 then you need to explain your conclusion, especially that your results (Table 2) and your Discussion ("However, it can be seen in this study that the respondents' awareness of the importance of the TT vaccine is very high") referred to the high TT vaccine coverage rates.   

- Thank you, we mean gaps among women aged 15-49 years. We have revised the sentences. "However, it can be seen in this study that the respondents' awareness of the importance of the TT vaccine is very high" referred to the high TT vaccine coverage rates in Bali and Nusa Tenggara regions.

Lines 2-3: “Indonesia as a whole, which is an archipelago, is one of the considerations and constraints involved in the coverage of the TT vaccine among women”, you need to clarify this point a little.        

- Thank you, we have clarified it.

# Peer review: Reviewer response for version 1

**DOI:** 10.5256/f1000research.56345.r87930

**Published:** 2021-08-09

**Authors:** Muhammad Hardhantyo

**Affiliations:** 1 Faculty of Health Science, Universitas Respati Yogyakarta, Yogyakarta, Indonesia; 2 Center for Health Policy and Management, Faculty of Medicine, Public Health and Nursing, Universitas Gadjah Mada, Yogyakarta, Indonesia

1. Please clarify the sudden increase of pregnant TT vaccine coverage in Indonesia from 2007 to 2008. It was mentioned that the vaccine coverage was 26% in 2007 and significantly increased to 65.2% in 2008. The strategies might benefit to improve the coverage in reproductive age women vaccination also.
2. Please determine the framework or theoretical model to decide the independent variables used in the study.
3. How was the categorization of household wealth determined by the authors of this article?
4. Please clarify the sampling method used in the articles, in the sample section the authors managed to reach **36,028** women aged 15– 49 years **who have not received the TT vaccine in Indonesia**. However, in the results section, the authors found that **the coverage of the TT vaccine** in Indonesia reached **75.32% out of the 36,028** respondents. Both statements were contradictory.
5. Please explain the process of handling the missing data and whether these might cause a bias in the estimation of parameters.
6. To reduce the number of tables, Tables 1 and 2 could be combined together.
7. Need to add 1 row to correct the position of the p-value and 95% Confidence Interval column in Table 4.
8. Underprivileged communities such as poor, low-educated women, single parents did not have any health insurance and the unemployed were less likely to have received TT vaccination compared to their counterparts. However, an interesting finding in the rural area where rural women are more likely to have received TT vaccination. Authors need to find more explanations and references.
9. Please provide reference to this statement " *The information obtained by the rural residents tends to be more centralized and there is no intervention from other sources such as the internet and minimizing false news; the information will be centered on the doctors, nurses and midwives visited at the health facility as a result. This is in contrast to the urban residents who prefer to obtain their health-related information independently. They tend to compare the results of the information obtained from the internet with that of the doctors, nurses, and midwives at the health facilities*."
10. In the Discussion section, please have a comment on any current public health efforts from the Indonesian government to increase immunization coverage?

Is the work clearly and accurately presented and does it cite the current literature?

Yes

If applicable, is the statistical analysis and its interpretation appropriate?

Yes

Are all the source data underlying the results available to ensure full reproducibility?

Yes

Is the study design appropriate and is the work technically sound?

Yes

Are the conclusions drawn adequately supported by the results?

Yes

Are sufficient details of methods and analysis provided to allow replication by others?

Partly

Reviewer Expertise:

Health Policy, Epidemiology, Child Health, Maternal Health

I confirm that I have read this submission and believe that I have an appropriate level of expertise to confirm that it is of an acceptable scientific standard, however I have significant reservations, as outlined above.

# Response

**Published:** 2021-08-22

**Authors:** Hidayat Arifin

**Affiliations:** Universitas Padjadjaran, Indonesia

Dear Muhammad Hardhantyo, PhD. 

 Thank you very much for the comments and suggestions. Here we put some revisions based on your suggestions. 

 1. Please clarify the sudden increase of pregnant TT vaccine coverage in Indonesia from 2007 to 2008. It was mentioned that the vaccine coverage was 26% in 2007 and significantly increased to 65.2% in 2008. The strategies might benefit to improve the coverage in reproductive age women vaccination also.

- Thank you for the suggestion. The difference in coverage from the highest and lowest during the period was possible due to differences in perceptions of the operational definition, problems in recording, reporting formats, and others. So there needs to be an effort to organize a recording and reporting system for pregnant women TT vaccination.

2. Please determine the framework or theoretical model to decide the independent variables used in the study.

- Thank you for the suggestion. The independent variables in this study were obtained from similar previous research variables, then adjusted to the demographic data in Indonesia.

3. How was the categorization of household wealth determined by the authors of this article?

- The wealth quintiles were divided into five categories based on principal component analysis (PCA). The wealth quintiles were measured by the percentage distribution of the de jure population using the wealth quintiles and the Gini coefficient. For the percentage distribution, the numerators were divided by the denominators multiplied by 100. The results were then divided into five equal parts from quintile one (poorest) to quintile five (richest); each included 20% of the total population. Then, it was categorized into the poorest, poorer, middle, richer, and richest respectively.

4. Please clarify the sampling method used in the articles, in the sample section the authors managed to reach 36,028 women aged 15– 49 years who have not received the TT vaccine in Indonesia. However, in the results section, the authors found that the coverage of the TT vaccine in Indonesia reached 75.32% out of the 36,028 respondents. Both statements were contradictory.

- Thank you for the correction. We have mistaken the sentences. We reached 36,028 women aged 15– 49 years who have and have not received the TT vaccine in Indonesia.

5. Please explain the process of handling the missing data and whether these might cause a bias in the estimation of parameters.

- In the beginning, we weighted the data set to make sure the equal portion in each province of Indonesia. Then, we used syntax “keep if” in STATA to exclude the missing data from the analysis. 

6. To reduce the number of tables, Tables 1 and 2 could be combined together.

- We combined Table 1 and Table 2.

7. Need to add 1 row to correct the position of the p-value and 95% Confidence Interval column in Table 4.

- Yes, we have added the suggestion. 

8. Underprivileged communities such as poor, low-educated women, single parents did not have any health insurance and the unemployed were less likely to have received TT vaccination compared to their counterparts. However, an interesting finding in the rural area where rural women are more likely to have received TT vaccination. Authors need to find more explanations and references.

- Thank you for the suggestion. “Number of midwives in rural to remote areas facilitate access for pregnant women to check up pregnancy, including the TT vaccine”.

9. Please provide reference to this statement "The information obtained by the rural residents tends to be more centralized and there is no intervention from other sources such as the internet and minimizing false news; the information will be centered on the doctors, nurses and midwives visited at the health facility as a result. This is in contrast to the urban residents who prefer to obtain their health-related information independently. They tend to compare the results of the information obtained from the internet with that of the doctors, nurses, and midwives at the health facilities."

- Thank you for the suggestion. We have added the references. "The information obtained by the rural residents tends to be more centralized and there is no intervention from other sources such as the internet and minimizing false news; the information will be centered on the doctors, nurses and midwives visited at the health facility as a result. This is in contrast to the urban residents who prefer to obtain their health-related information independently. They tend to compare the results of the information obtained from the internet with that of the doctors, nurses, and midwives at the health facilities."

10. In the Discussion section, please have a comment on any current public health efforts from the Indonesian government to increase immunization coverage?

- Thank you for the suggestion. The Indonesian government has many efforts to increase the TT vaccination coverage, such as equitable availability of midwives in rural to remote areas and regulations that require brides to be vaccinated TT according to government regulation number 02 of 1989 about Tetanus Toxoid Immunization before marriage.

# Peer review: Reviewer response for version 1

**DOI:** 10.5256/f1000research.56345.r87631

**Published:** 2021-07-23

**Authors:** Heri Kuswanto

**Affiliations:** 1 Center for Disaster Mitigation and Climate Change, Institut Teknologi Sepuluh Nopember, Surabaya, Indonesia

This paper has been written very well, with a structure that is easily understood. However, there is a minor comment that might be considered to improve the quality of the paper:

 The interpretation of the logistic regression model is done partially without trying to interrelate the finding on another variable. In this paper, since the authors did not conduct a collinearity test, some findings need to be clarified further. For instance, I guess women in rural areas are mostly without insurance, but the statistical test showed that either women in rural areas or women with insurance are likely to be vaccinated. This finding seems to contradict each other which might be caused by a high degree of correlation between variables (considering the fact that the odds ratio is also very close to 1). If it is difficult to be done, I would suggest that the authors state the assumption of no significant correlation among predictors.

Is the work clearly and accurately presented and does it cite the current literature?

Yes

If applicable, is the statistical analysis and its interpretation appropriate?

Yes

Are all the source data underlying the results available to ensure full reproducibility?

Yes

Is the study design appropriate and is the work technically sound?

Yes

Are the conclusions drawn adequately supported by the results?

Yes

Are sufficient details of methods and analysis provided to allow replication by others?

Yes

Reviewer Expertise:

Statistical data analysis, econometrics

I confirm that I have read this submission and believe that I have an appropriate level of expertise to confirm that it is of an acceptable scientific standard.

# Response

**Published:** 2021-08-22

**Authors:** Hidayat Arifin

**Affiliations:** Universitas Padjadjaran, Indonesia

Dear Prof. Heri Kuswanto

 Thank you very much for your comments and suggestion to improve the quality of our manuscript. we have added in the limitations of the study that there is the assumption of no significant correlation among predictors.
